# Photoelectrochemical hydrogen production in water using a layer-by-layer assembly of a Ru dye and Ni catalyst on NiO†
†Electronic supplementary information (ESI) available. See DOI: 10.1039/c6sc00715e. Additional data related to this publication are available at the University of Cambridge data repository (https://www.repository.cam.ac.uk/handle/1810/255990).


**DOI:** 10.1039/c6sc00715e

**Published:** 2016-05-09

**Authors:** Manuela A. Gross, Charles E. Creissen, Katherine L. Orchard, Erwin Reisner

**Affiliations:** a Christian Doppler Laboratory for Sustainable SynGas Chemistry , Lensfield Road , CB2 1EW Cambridge , UK . Email: reisner@ch.cam.ac.uk

## Abstract

Capture and conversion of sunlight into the storable energy carrier H_2_ can be achieved through photoelectrochemical water splitting using light-absorbing cathodes and anodes bearing H_2_ and O_2_ evolving catalysts. Here, we report on the development of a dye-sensitised p-type nickel oxide (NiO) photocathode with a hexaphosphonated Ru(2,2′-bipyridine)_3_ based dye (**RuP3**) and a tetraphosphonated molecular [Ni(P_2_N_2_)_2_]^2+^ type proton reduction catalyst (**NiP**) for the photoreduction of aqueous protons to H_2_. A layer-by-layer deposition approach was employed, using Zr^4+^ ions to link the phosphonate units in **RuP3** and **NiP** in a supramolecular assembly on the NiO photocathode. This approach keeps the dye in close proximity to the catalyst and semiconductor surface, but spatially separates **NiP** from NiO for advantageous electron transfer dynamics. The NiO|**RuP3**–Zr^4+^–**NiP** electrodes generate higher photocurrents and are more stable than photocathodes with **RuP3** and **NiP** co-immobilised on the NiO surface in the absence of Zr^4+^ cations linking dye and catalyst. The generation of H_2_ with the NiO|**RuP3**–Zr^4+^–**NiP** hybrid electrode in pH 3 aqueous electrolyte solution during irradiation with a UV-filtered solar light simulator (*λ* > 400 nm, 100 mW cm^–2^, AM1.5G) has been confirmed by gas chromatography at an underpotential of 300 mV (*E*_appl_ = +0.3 V *vs.* RHE), demonstrating the potential of these electrodes to store solar energy in the chemical bond of H_2_.

## Abstract 2

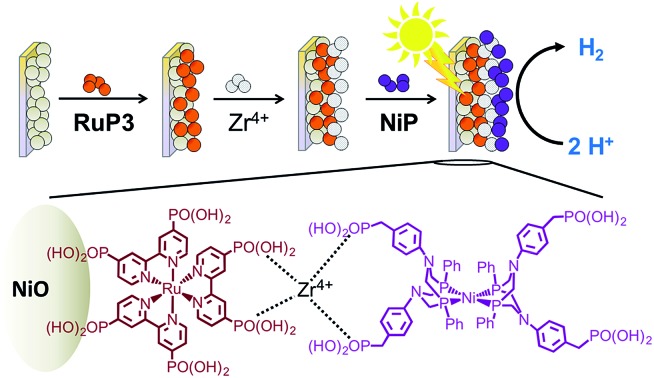
Layer-by-layer assembly of a Ru dye and Ni catalyst on a p-type NiO photocathode enables photoelectrochemical H_2_ generation in water.

## Introduction

Photovoltaic technology, such as dye-sensitised solar cells, enables the conversion of solar energy into electricity and is commercially available.^1^ Solar fuel devices allow for the storage of solar energy in a chemical energy carrier, but are only in an early stage of development and have not yet reached the required efficiencies and stabilities at low cost suitable for application.^2^ Splitting of water into its elements in a photoelectrochemical (PEC) cell is a potentially sustainable means of generating renewable H_2_.^3^ However, a lack of novel strategies to electronically couple a light-harvesting unit to a stable proton reduction catalyst is currently holding back advances in the development of high-performance PEC devices.^4^ Molecular dyes and catalysts offer several advantages over heterogeneous alternatives as their composition and activity can be more easily synthetically controlled and fine-tuned.^5^ They provide an excellent platform to develop rational strategies such as layer-by-layer deposition^6^ to integrate dyes and catalysts on electrode materials and enable detailed mechanistic studies to improve our understanding of photoelectrode assemblies.^7^ Ultimately, these insights may contribute to the establishment of advanced approaches to fabricate electrodes in PEC water splitting cells.

We have previously reported on several suspension-based photocatalytic H_2_-generation systems with molecular catalysts attached to light-absorbing nanoparticles to advance our understanding of efficient combination of dyes and catalysts on semiconducting surfaces.^8^ However, all of these systems relied on the use of a sacrificial electron donor (SED) to provide the reducing equivalents for fuel formation. SEDs are chemicals of relatively high value and are consumed stoichiometrically during the H_2_ generation process. They are disadvantageous as half-reaction promoting agents as they often form unknown decomposition products *via* highly reactive intermediates, which can interfere with the catalytic system, and should ultimately be avoided. One approach to bypass the need for a SED in a H_2_ evolution system is to immobilise the catalyst onto a photocathode material that can be incorporated into a PEC cell. The redox cycle can then be closed by use of a suitable (photo)anode for water/substrate oxidation.3b,c One of the main challenges for the assembly of molecule-based PEC devices is the design and effective integration of H_2_ evolving electrocatalysts and dyes onto the electrodes, which should ultimately allow for efficient solar light absorption, charge separation and chemical catalysis.

Photocathode materials that have been studied for solar H_2_ generation include p-silicon,^9^ p-GaP^10^ and p-NiO.^11^ Of these, p-type NiO offers the possibility to produce nanostructured high-surface area electrodes without the need for specialised techniques. High roughness is a prerequisite for maximising dye and catalyst loading on a given geometric surface area. Dye-sensitised NiO photocathodes in organic or aqueous electrolyte solution have been studied with sacrificial electron acceptors (SEAs),^12^ [FeFe]-hydrogenase mimics,^13^ and rhodium and cobaloxime catalysts.11b–e,^14^ While charge transfer and recombination kinetics of NiO|dye^15^ and NiO|dye–catalyst^13^ assemblies have been studied in detail with time-resolved spectroscopy, light-driven H_2_ production with dye-sensitised NiO photocathodes remains challenging and only cobaloxime-based catalysts have been reported to generate H_2_ with NiO.11b,d,e The binding of the cobaloxime catalyst in these cases was achieved through a linker tethered to NiO bearing a free pyridine moiety, which coordinates to the axial coordination sphere of the cobaloxime.11c–e Axial pyridine units coordinated to cobaloximes have been shown to be inherently labile during catalytic turnover,5b,10b,^16^ and therefore H_2_ evolution catalysts with a stable ligand framework are needed.

**Fig. 1:**
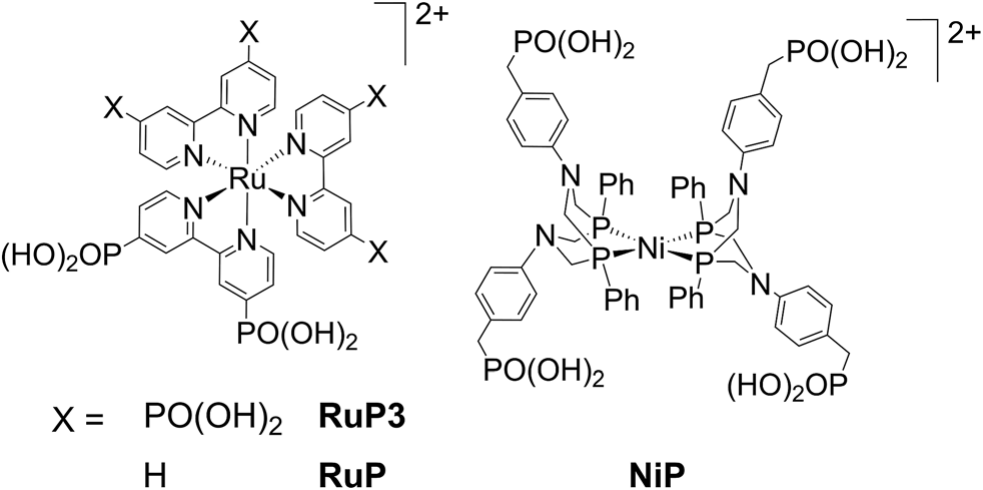
Structures of the dyes (**RuP** and **RuP3**) and proton reduction catalyst (**NiP**) used in this study. Chloride (**RuP3**) and bromide (**RuP**, **NiP**) counter-ions are omitted for clarity.

Nickel bis(diphosphine) type catalysts, [Ni(P_2_N_2_)_2_]^2+^, are attractive proton reduction catalysts as they operate in water,8b,^17^ and display higher activity than cobaloximes in photocatalytic H_2_ production.17a,^18^ The [Ni(P_2_N_2_)_2_]^2+^ complex also provides a stable ligand framework with no coordinating moieties becoming labile during the catalytic cycle,^19^ and is therefore less susceptible to catalyst dissociation from a surface due to ligand dissociation.^20^ [Ni(P_2_N_2_)_2_]^2+^ catalysts have previously been shown to retain high electrocatalytic activity for H_2_ generation from water when immobilised on multi-walled carbon nanotubes (CNTs) *via* covalent linking^20^ or π–π interactions of pyrene moieties,^21^ and when immobilised *via* phosphonate groups on mesostructured TiO_2_ electrodes.^22^ The phosphonated catalyst, **NiP** (Fig. 1), was also used as catalyst on TiO_2_ nanoparticles sensitised with a phosphonated ruthenium-based dye (**RuP**, Fig. 1) in photocatalytic H_2_ generation with a SED.17*a* A [Ni(P_2_N_2_)_2_]^2+^ and a [Ni(PNP)_2_]^2+^ type catalyst have previously been immobilised on p-Si,9c,^23^ but photocatalytic H_2_ generation was only shown for the latter assembly in acetonitrile with trifluoroacetic acid as proton source. Thus, previous work on H_2_ generation with [Ni(P_2_N_2_)_2_]^2+^ catalysts relied on the use of a SED, an applied electrochemical overpotential or non-aqueous conditions to generate H_2_.

**Fig. 2:**
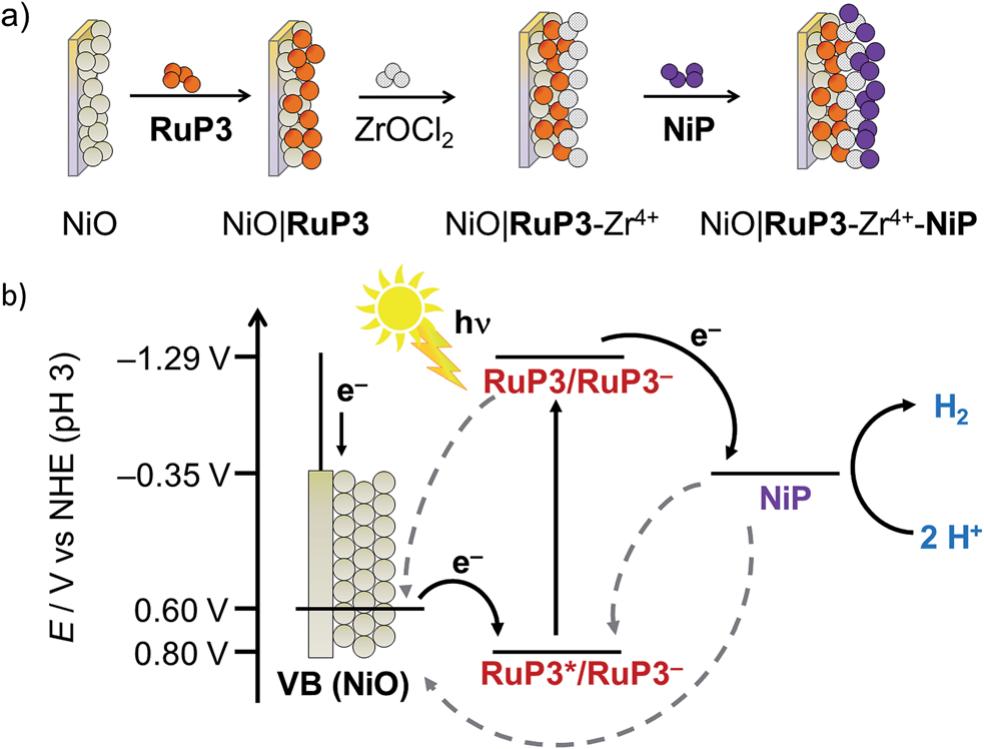
(a) Supramolecular dye–catalyst assembly on photocathode: layer-by-layer deposition of **RuP3**, Zr^4+^ and **NiP** on p-type NiO photocathode (see text for details). (b) Energy diagram of the NiO photocathode assembly showing the proposed electron transfer mechanisms (reductive quenching) as black solid arrows and possible recombination pathways as grey dashed arrows between p-NiO, **RuP3** and **NiP**.

In this study, PEC H_2_ evolution was investigated on a p-type NiO photocathode sensitised with a hexaphosphonated ruthenium tris(bipyridine) based dye (**RuP3**, Fig. 1)^24^ and **NiP**. A rational and simple procedure of sequential immersion of NiO films into solutions of **RuP3**, ZrOCl_2_ and **NiP** was employed to produce electrodes of the type NiO|**RuP3**–Zr^4+^–**NiP** (Fig. 2),^25^ where the catalyst is spatially separated from the semiconductor electrode to reduce inefficiencies from charge-recombination.^13^ Our study shows that increasing the distance of the catalyst from the NiO surface results in substantially enhanced PEC performance in terms of photocurrent and stability when compared to NiO|**RuP3**–**NiP**, where the dye and catalyst are co-immobilised on NiO. We demonstrate a dye-sensitised NiO hybrid electrode with an adsorbed Ni catalyst, which has the potential to store solar energy in the bond of H_2_ without the decomposition of valuable sacrificial agents or an applied overpotential. Rational layer-by-layer assembly of dye and catalyst on a semiconductor electrode is therefore established as a route to produce functional PEC H_2_ production systems.

## Results and discussion

### Assembly and pre-catalysis characterisation

Mesoporous NiO was grown on tin-doped indium oxide (ITO)-coated glass electrodes by a previously reported hydrothermal method^26^ from a solution containing Ni(NO_3_)_2_·6H_2_O and hexamethylenetetramine (0.25 M each). NiO electrodes were sensitised by immersion in a **RuP3** solution (1 mM in H_2_O) overnight to give NiO|**RuP3** for PEC experiments. Immobilisation of monolayers of molecular compounds *via* phosphonic acids on metal oxide surfaces is a well-established tool for surface modification.25*a***NiP** was immobilised on the NiO|**RuP3** photocathodes in a layered assembly using Zr^4+^ to link the phosphonates of dye and catalyst (Fig. 2; see below for more details). Zr^4+^ ions bind strongly to phosphonic acid moieties and were previously used for creating multi-layered Zr^4+^-phosphonate structures.^25^,^27^ Zr^4+^ was bound by dipping NiO|**RuP3** electrodes into a solution of ZrOCl_2_ (5 mM in EtOH/H_2_O, 60/40 v/v) for 2 h, rinsing with H_2_O and EtOH and drying under a stream of N_2_. The NiO|**RuP3**–Zr^4+^ electrodes were subsequently submerged in a solution of **NiP** (0.5 mM in MeOH) for at least 2 h to obtain the supramolecular NiO|**RuP3**–Zr^4+^–**NiP** electrode assembly with one layer of dye and one layer of catalyst linked by Zr^4+^. All immobilisation steps were carried out at room temperature.

In previously reported homogeneous, SED-promoted photocatalytic H_2_ generation systems, the ratio between the dye and the catalyst was found to strongly influence the overall performance.17a,^28^ We have therefore varied the ratio of co-immobilised **RuP3** and **NiP** on the p-NiO photocathodes. The layer-by-layer deposition approach allowed the **RuP3** and **NiP** loading on the metal oxide surface to be controlled more precisely, creating Zr^4+^-linked dye/catalyst layers by repeated immobilisation cycles.25*c* Repeated deposition of Zr^4+^ and either **RuP3** dye or **NiP** catalyst gave electrodes of the structure NiO|(**RuP3**–Zr^4+^)_2_–**NiP** (containing two layers of **RuP3**–Zr^4+^) or NiO|**RuP3**–(Zr^4+^–**NiP**)_2_ (containing two layers of Zr^4+^–**NiP**). For control experiments, NiO|**RuP3** electrodes were submerged directly in **NiP** solution without prior immobilisation of Zr^4+^ ions, and the so-obtained NiO|**RuP3**–**NiP** electrodes were also studied. Additionally, **RuP3** was replaced with the diphosphonated dye **RuP** (Fig. 1), which only allows binding to NiO and does not offer any phosphonic acid moieties to generate Zr^4+^-linked phosphonate networks.

**Fig. 3:**
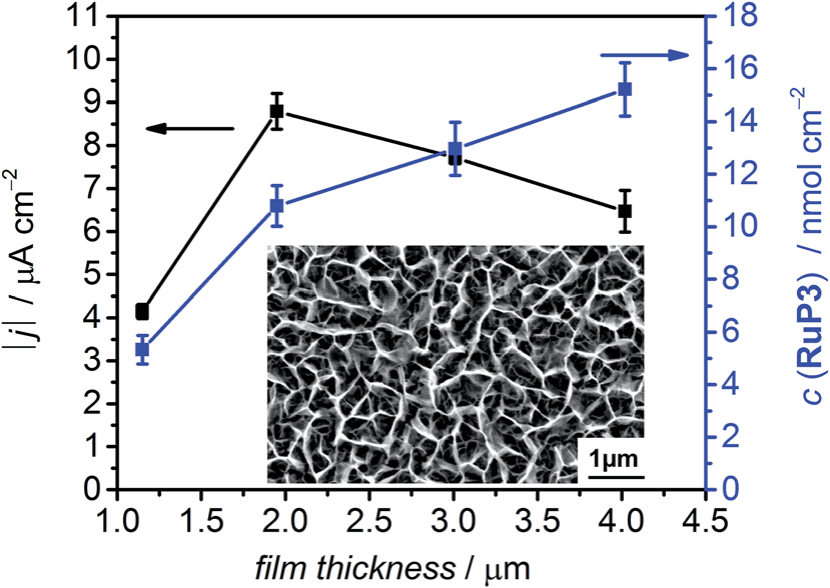
Photocurrent response of NiO|**RuP3** under visible light irradiation (*λ* > 400 nm, AM1.5G filter, 100 mW cm^–2^) after 100 s at *E*_appl_ = 0.3 V *vs.* RHE in Na_2_SO_4_ (0.1 M, pH 3) in the presence of 4,4′-dithiodipyridine (DTDP, 1 mM) as SEA and dye loading (no applied potential; cm^–2^ refers to the geometrical surface area of the NiO electrode) with varying thicknesses of the NiO films. Inset: SEM image of optimised p-type NiO electrode (2 μm film thickness).

The photocurrent response of NiO|**RuP3** electrodes with different NiO film thickness was recorded in a PEC experiment in aqueous electrolyte solution (0.1 M Na_2_SO_4_, pH 3) in the presence of the SEA 4,4′-dithiodipyridine (DTDP, 1 mM; Fig. 3 and S1†) at room temperature. DTDP is reduced in two irreversible steps, with the first reduction occurring at *E*_p_ = –0.06 V *vs.* the reversible hydrogen electrode (RHE). Thickness, morphology and crystallinity of the NiO films was studied by varying the reaction conditions (see Table S1 and Fig. S2 and S3† for powder X-ray diffraction pattern and scanning electron microscopy images). The optimised films have an average pore size (measured as distance between sheets) of 290 ± 81 nm and a film thickness of 2 μm (Fig. 3). The flatband potential of this NiO electrode was determined *via* electrochemical impedance spectroscopy at 0.75 V *vs.* RHE from Mott–Schottky analysis (Fig. S4†) and shows Nernstian pH dependence (one electron/one proton couple) in aqueous electrolyte solution, which is in agreement with previous reports on NiO electrodes.^29^

The photocurrent response of NiO|**RuP3** and the loading capacity for **RuP3** on NiO films with different film thickness was studied by PEC methods and desorption of the attached dye with 0.1 M NaOH and monitoring the UV-vis absorption at *λ* = 295 nm in solution (Table S1†). The dye loading capacity increased as expected with increasing film thickness, but the highest photocurrent density was achieved with a 2 μm thick NiO film, despite the higher dye loadings for thicker films (Fig. 3, Table S1†). This observation is consistent with the known limitation of hole mobility and lifetime in NiO,^30^ which suggests that the photocurrent generated with **RuP3**-sensitised NiO photocathodes is a balance between limiting charge transport in thick films and dye loading on thin films.

We subsequently investigated the interface between molecular components and the p-type NiO surface of the NiO|**RuP3**–Zr^4+^–**NiP** electrodes by UV-vis (Fig. S5†), attenuated total reflection infrared (ATR-IR; Fig. S6†), X-ray photoelectron (XPS; Fig. S7†) and inductively coupled plasma-optical emission spectroscopies (ICP-OES). The UV-vis spectrum of a bare NiO thin film shows the typical strong absorption at *λ* < 380 nm, and also a broad indirect transition at *λ* < 550 nm.^29^ All **RuP3**-sensitised NiO electrodes exhibit the typical MLCT absorption band for Ru(bpy)_3_-based dyes around *λ* = 465 nm and strong π-π* absorption in the UV region.^24^ When **NiP** is present on the electrodes a small shoulder at *λ* = 520 nm could be observed, which is typical for the d–d transition in **NiP** as also observed in solution spectra (Fig. S5†).

The ATR-IR spectra of the NiO|**RuP3** electrodes at *ν* = 800 to 1250 cm^–1^ in the presence and absence of Zr^4+^ and **NiP** are shown in Fig. S6.† This region is characteristic for P=O and P–O–R vibrations, which are also observed in the spectra of powdered **RuP3**, **RuP** and **NiP**.^31^ NiO|**RuP3** electrodes show a broad band at *ν* ∼ 1100 cm^–1^ attributed to P=O vibrations in **RuP3**, which is absent in a bare NiO film. In a NiO|**RuP3**–Zr^4+^ electrode, a small additional IR band at *ν* ∼ 983 cm^–1^ appears when compared to NiO|**RuP3**, and the signal increases and shifts to slightly higher wavenumbers for NiO|**RuP3**–Zr^4+^–**NiP** (*ν* ∼ 986 cm^–1^) and NiO|**RuP3**–(Zr^4+^–**NiP**)_2_ (*ν* ∼ 992 cm^–1^). This shift is in good accordance with previously reported values for Zr^4+^ bound to phosphonic acid groups.25*b*

XPS analysis was used to further characterise the modified NiO surface, and the presence of Ru and Zr on NiO after each layering step was confirmed (Fig. S7†). The signal for **NiP** could not be distinguished from the NiO background signal. Instead, mesoporous indium-tin oxide (ITO, 3.3 μm thickness) electrodes were prepared according to a previously reported procedure^32^ and used here as a scaffold. The XPS signals of ITO|**RuP3**–**NiP**, ITO|**RuP3**–Zr^4+^ and ITO|**RuP3**–Zr^4+^–**NiP** electrodes (prepared analogously to the related NiO hybrids) were analysed. In the presence of **NiP**, peaks at 855.0 and 872.5 eV were observed and were assigned to the Ni 2p_3/2_ and Ni 2p_1/2_ of the molecular **NiP**, respectively.17c,^33^ The P 2p signal for ITO|**RuP3**–**NiP** and ITO|**RuP3**–Zr^4+^–**NiP** electrodes is broad (133.0–131.0 eV) and consists of signals for the phosphonic acid bound to ITO^34^ and for the phosphine ligand.^33^ In contrast, ITO|**RuP3**–Zr^4+^ showed only a sharp XPS peak at 133.5 eV, which is assigned to the –PO(OH)_2_ groups.^34^ As the **RuP3**–Zr^4+^–**NiP** was assembled in identical manner on ITO and NiO, the presence of intact **NiP** on ITO supports its integrity on NiO prior to catalysis.

Finally, we quantified the immobilised dye and catalyst on NiO by UV-vis spectroscopy and ICP-OES analysis after desorption in aqueous NaOH (0.1 M). The overlap of **RuP3** and **NiP** in the electronic absorption spectra prevented the simultaneous determination of dye and catalyst loading on the electrodes and ICP-OES measurements were used instead. Quantification of **NiP** on NiO was challenging due to the high nickel background from the electrode. Mesoporous ITO electrodes were used again as a scaffold and analysis of ITO|**RuP3**, ITO|**RuP3**–**NiP**, ITO|**RuP3**–Zr^4+^ and ITO|**RuP3**–Zr^4+^–**NiP** electrodes was carried out. The quantity of **NiP** on NiO was estimated from the Ru : Ni ratio found on the ITO electrodes (Table 1). The ICP-OES data for the amount of **RuP3** immobilised on bare NiO (10.7 ± 0.3 nmol cm^–2^) correlates well with quantification by UV-vis spectroscopy after dye desorption (10.8 ± 0.8 nmol cm^–2^). Analysis of ITO|**RuP3**–**NiP** electrodes revealed a Ru : Ni ratio of 2.6 ± 0.04 : 1 and this ratio decreases only slightly to ∼2.2 ± 1.5 : 1 on ITO|**RuP3**–Zr^4+^–**NiP**, indicating that a similar amount of **NiP** is immobilised in the presence and absence of Zr^4+^. This ratio allows us to estimate a **NiP** loading of 4.98 ± 3.54 nmol cm^–2^ for NiO|**RuP3**–Zr^4+^–**NiP**, which is comparable to the loading of a cobaloxime catalyst on CdSe-sensitised NiO (∼3 nmol cm^–2^ for a 5.6 μm thick NiO film).11*b*

**Table 1:** Quantification of **RuP3** and **NiP** on the NiO electrodes (determined by ICP-OES) and photocurrents (AM1.5G filter, 100 mW cm^–2^ and *λ* > 400 nm) of the respective electrodes after 100 s CPPE at *E*_appl_ = 0.3 V *vs.* RHE at room temperature. **RuP3** and **NiP** loading on ITO is also shown. Loading concentrations are reported for the geometrical surface area of the electrodes.^*a*^

Composition	**RuP3**/nmol cm^–2^	**NiP**/nmol cm^–2^	|*j*|/μA cm^–2(a)^	|*j*|/μA cm^–2(b)^
NiO|**RuP3**	10.7 ± 0.3	—	0.51	3.0
NiO|**RuP3**–**NiP**	9.4 ± 1.6	3.57 ± 0.61^(c)^	0.94	n.d.^(d)^
NiO|**RuP3**–Zr^4+^	6.3 ± 2.2	—	0.76	7.62
NiO|**RuP3**–Zr^4+^–**NiP**	6.3 ± 3.1	4.98 ± 3.54^(c)^	6.40	14.0
NiO|**RuP3**–(Zr^4+^–**NiP**)_2_	n.d.	n.d.	8.82	n.d.
NiO|(**RuP3**–Zr^4+^)_2_–**NiP**	n.d.	n.d.	2.18	n.d.
ITO|**RuP3**–**NiP**	37.6 ± 0.2	14.3 ± 0.2	—	—
ITO|**RuP3**–Zr^4+^	53.2 ± 20.5	—	—	—
ITO|**RuP3**–Zr^4+^–**NiP**	41.3 ± 20.3	19.2 ± 9.9	—	—

^*a*^Measured in ^(a)^the absence and ^(b)^presence of DTDP as SEA. ^(c)^Calculated value from **RuP3** : **NiP** ratio on ITO. ^(d)^Not determined.

### Photoelectrochemical experiments

**Fig. 4:**
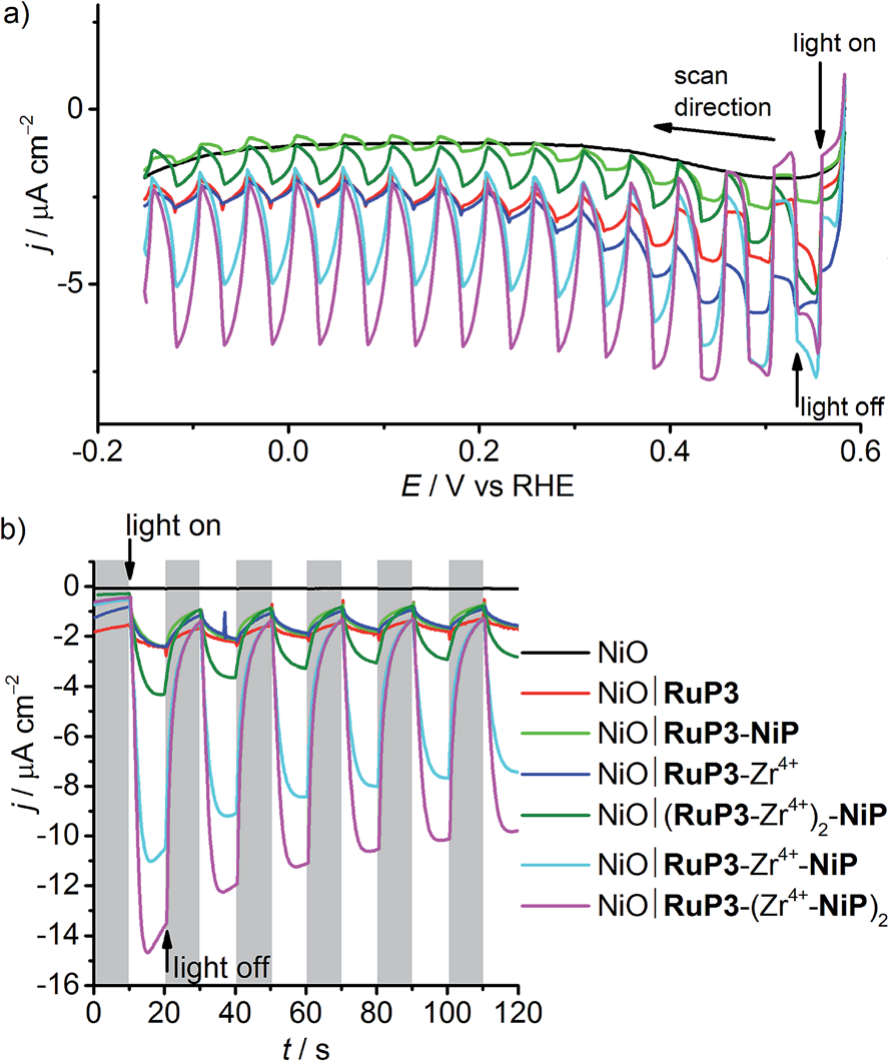
(a) LSV scans of NiO electrodes at a scan rate of 5 mV s^–1^ under chopped light irradiation (*λ* > 400 nm, AM1.5G filter, 100 mW cm^–2^). (b) Chronoamperometry of NiO working electrodes under chopped light irradiation (10 s on/off cycles) at *E*_appl_ = 0.3 V *vs.* RHE. All experiments were performed in a 3-electrode setup with a Ag/AgCl/KCl_(sat.)_ reference and a Pt mesh counter electrode under N_2_ in a custom-made one-compartment PEC cell at room temperature.

The photocathodes were studied in PEC experiments in aqueous electrolyte solution (0.1 M Na_2_SO_4_, pH 3) in a three-electrode setup with a Ag/AgCl reference and a Pt counter electrode at room temperature. Linear sweep voltammetry (LSV) scans were recorded between *E* = +0.6 V and –0.15 V *vs.* RHE at a scan rate of 5 mV s^–1^ in the dark and under chopped and UV filtered irradiation with simulated solar light (*λ* > 400 nm, 100 mW cm^–2^, AM1.5G; Fig. 4a). All NiO electrodes (irrespective of being loaded with molecules or not) displayed a partially reversible oxidation wave between *E* = 0.42 and 0.48 V *vs.* RHE, which is assigned to the Ni^3+^/Ni^2+^ redox couple (Fig. S8†).^29^,30b In the absence of dye, only marginal photocurrent densities (reported per geometrical surface area) were observed in the studied potential range. Transient and small cathodic photocurrents were observed upon irradiating NiO|**RuP3** (|*j*| ∼ 1.4 μA cm^–2^ at 0.5 V *vs.* RHE) and NiO|**RuP3**–Zr^4+^ (|*j*| ∼1.2 μA cm^–2^ at 0.5 V *vs.* RHE) electrodes, indicating hole injection of the excited dye, RuP3*, into the valence band of NiO (Fig. 2, for details see Mechanistic interpretation below). Quenching of RuP3* by NiO results in the formation of a reduced dye, **RuP3^–^**, which is then likely to undergo rapid charge recombination with the holes generated in NiO in absence of a suitable electron acceptor.^13^,15b A comparable photoresponse was observed between NiO|**RuP3** and NiO|**RuP3**–**NiP**, indicating that direct binding of the **NiP** to NiO results in an unproductive assembly with negligible electron accumulation at **NiP**. When a NiO|**RuP3**–Zr^4+^–**NiP** electrode was used, however, an initial photocurrent density up to |*j*| ∼ 5.56 μA cm^–2^ was achieved under the same experimental conditions (Fig. 4a).

Subsequently, the importance of the phosphonic acid moieties in the assembly of functional electrodes was investigated (Fig. S9†). When the diphosphonated dye **RuP** (Fig. 1)^35^ is immobilised on NiO instead of **RuP3**, no phosphonic acid binding sites are available for Zr^4+^. A comparable photocurrent was observed for a NiO|**RuP**–Zr^4+^–**NiP** and NiO|**RuP**–**NiP** electrode, which confirms that Zr^4+^ binding to the electrode assembly occurs *via* the free phosphonic acid groups of **RuP3** and not *via* intercalation in the mesoporous NiO scaffold or precipitation as ZrO_2_ during the deposition procedure. The results from LSV confirm that the presence of **RuP3**, **NiP** and Zr^4+^ in the layered assembly is essential for the functional hybrid photoelectrode.

Chronoamperograms for all photoelectrodes were recorded at an applied potential, *E*_appl_, of 0.3 V *vs.* RHE (Table 1 and Fig. 4b) and at *E*_appl_ = 0.5 V *vs.* RHE (Fig. S10†) under chopped light irradiation and confirmed the trends observed by LSV. Cathodic photocurrents for NiO|**RuP3** (|*j*| ∼ 0.5 μA cm^–2^) and for NiO|**RuP3**–Zr^4+^ (|*j*| ∼ 0.76 μA cm^–2^) were small at *E*_appl_ = 0.3 V *vs.* RHE. The photocurrent of a NiO|**RuP3**–**NiP** electrode (|*j*| = 0.94 μA cm^–2^) at *E*_appl_ = 0.3 V *vs.* RHE was more than six times smaller than the photocurrent achieved with a NiO|**RuP3**–Zr^4+^–**NiP** electrode (initial photocurrent |*j*| = 9.97 μA cm^–2^ stabilised to 6.40 μA cm^–2^ after 100 s).

The photoelectrodes were also investigated in the presence of DTDP as SEA. A UV-vis spectrum of **NiP** in solution in the presence and absence of DTDP (1 mM) shows no differences, confirming the stability of the catalyst in the presence of DTDP (Fig. S11†). As expected, the presence of a soluble SEA in the electrolyte solution enhanced the photocurrents observed for the NiO|**RuP3** (|*j*| = 3.0 μA cm^–2^) and NiO|**RuP3**–Zr^4+^ electrodes (|*j*| = 7.6 μA cm^–2^) at *E*_appl_ = 0.3 V *vs.* RHE (Fig. S12†). A significant increase in photocurrent was also found for a NiO∣**RuP3**–Zr^4+^–**NiP** electrode in the presence of DTDP in solution (|*j*| = 14.0 μA cm^–2^) compared to a SEA-free system (|*j*| = 6.4 μA cm^–2^). This suggests that surface-bound **NiP** is efficiently reduced by the generated photoelectrons and electron transfer from the reduced catalyst to the dissolved SEA in the electrolyte solution is faster than competing charge recombination. An increased photocurrent in the presence of DTDP suggests that the reduction of the SEA is faster than **NiP**-promoted catalysis.

The effect of the ratio of dye and catalyst was also studied by preparing electrodes with either two layers of dye, NiO|(**RuP3**–Zr^4+^)_2_–**NiP**, or two layers of catalyst, NiO|**RuP3**–(Zr^4+^–**NiP**)_2_. The photocurrent generated by a NiO|(**RuP3**–Zr^4+^)_2_–**NiP** electrode after 100 s chronoamperometry at *E*_appl_ = 0.3 V *vs.* RHE (|*j*| = 2.18 μA cm^–2^) was almost three times lower than the photocurrent of a NiO|**RuP3**–Zr^4+^–**NiP** electrode (Table 1). In contrast, approximately 40% increase in photocurrent was observed for a NiO|**RuP3**–(Zr^4+^–**NiP**)_2_ electrode (|*j*| = 8.82 μA cm^–2^). These experiments confirmed that there is a significant dependence of the number of layers on the photocurrent response and show that transient photocurrents generated by dye-sensitised NiO electrodes are dependent on the strategy used to co-immobilise dye and catalyst on the surface. This layer-by-layer approach has previously been successful for dye-sensitised photoanodes for water oxidation,^36^ but has not yet been explored for fuel-forming photocathodes.

**Fig. 5:**
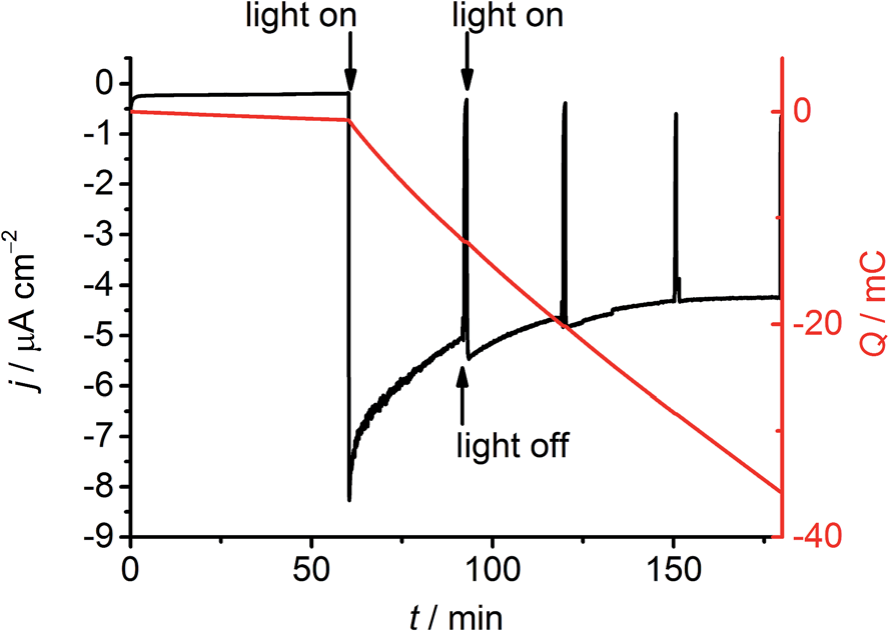
CPPE of a NiO|**RuP3**–Zr^4+^–**NiP** photoelectrode (1 cm^2^) at *E*_appl_ = 0.3 V *vs.* RHE in Na_2_SO_4_ (0.5 M, pH 3) at room temperature. Electrolysis was performed in a 2-compartment PEC cell with the working and counter compartment separated by a Nafion membrane. The working electrode was held in the dark for 1 h, followed by irradiation for 2 h using a solar light simulator (100 mW cm^–2^, AM1.5G filter, *λ* > 400 nm).

The formation of H_2_ at the NiO|**RuP3**–Zr^4+^–**NiP** electrode was then studied by controlled potential photoelectrolysis (CPPE; Fig. 5). Irradiation of the NiO|**RuP3**–Zr^4+^–**NiP** and NiO|**RuP3**–(Zr^4+^–**NiP**)_2_ electrodes at *E*_appl_ = 0.3 V *vs.* RHE in a two-compartment PEC cell generated 6.11 ± 0.68 and 6.8 ± 0.9 nmol H_2_ in the headspace (analysed by gas chromatography with N_2_ carrier gas) with a faradaic efficiency of 8.6 ± 2.3% and 10.1 ± 1.8%, respectively (Table S2†). Increasing the light intensity to 200 mW cm^–2^ or using an *E*_appl_ = 0.5 V *vs.* RHE did not alter the H_2_ production rate nor improve the faradaic efficiency for NiO|**RuP3**–Zr^4+^–**NiP**. In the absence of **NiP** or in the dark, no H_2_ generation was observed. NiO|**RuP3**–**NiP** and NiO|(**RuP3**–Zr^4+^)_2_–**NiP** electrodes produced only traces of H_2_ below the limit of quantification (LOQ) of the thermal conductivity detector. The low activity of the NiO|**RuP3**–Zr^4+^–**NiP** electrode is likely due to inefficient charge transfer dynamics at the NiO|**RuP3** interface and between reduced **NiP** and oxidised **RuP3** or NiO (Fig. 2b). Nevertheless, confirmation of H_2_ evolution at the photocathode is an important step towards the goal of implementing dye-sensitised NiO photocathodes in full water splitting PEC cells.11c,^13^

### Post-catalysis characterisation

The electrode stability was further examined by ICP-OES analysis of the Ru content in both electrolyte solution and on the electrodes following 2 h CPPE at *E*_appl_ = 0.3 V *vs.* RHE. For NiO|**RuP3**–Zr^4+^–**NiP** a substantial amount of the initially loaded Ru (6.3 ± 3.1 nmol cm^–2^) was recovered from the used electrodes (3.4–7.4 nmol Ru cm^–2^) upon desorption in aqueous NaOH (0.1 M). Almost no Ru leaked into the electrolyte solution of the working or counter compartments (<0.2 nmol total Ru content). The opposite was observed for the NiO|**RuP3**–**NiP** electrodes after 2 h CPPE and 4.9–5.3 nmol total Ru content were found in the electrolyte solution of the working compartment and very little Ru (0.39–0.74 nmol cm^–2^) was recovered from the used working electrode. The Zr^4+^ interlayer might contribute to stabilising **RuP3** on the surface of NiO electrodes under CPPE conditions. The stability of the NiO|**RuP3**–Zr^4+^–**NiP** working electrodes against material degradation was also confirmed by the lack of observable changes in the cyclic voltammograms before and after CPPE (Fig. S13†) and a significant alteration for NiO|**RuP3**–Zr^4+^ electrodes after CPPE at 0 V *vs.* RHE (Fig. S14†), indicating material instability of the latter.

The XPS spectra of a NiO|**RuP3**–Zr^4+^–**NiP** electrode were recorded and confirmed the presence of both **RuP3** (281 eV, Ru 3d_5/2_) and Zr^4+^ (182.6 and 185 eV for Zr 3d_5/2_ and Zr 3d_3/2_, respectively) before and after 2 h CPPE at *E*_appl_ = 0.3 V *vs.* RHE (Fig. S7†). No new peaks in the Ru 3d_5/2_ region were observed after CPPE to indicate formation of Ru/RuO_*x*_ particles. The presence and integrity of **NiP** on the NiO|**RuP3**–Zr^4+^–**NiP** electrode after CPPE could not unambiguously be confirmed by XPS due to the presence of the NiO. However, the stability of **NiP** on the electrode surface of a mesostructured TiO_2_ electrode under reducing conditions (*E*_appl_ = –0.25 V *vs.* RHE) in the dark in otherwise identical aqueous electrolyte solution (0.1 M Na_2_SO_4_, pH 3) has previously been confirmed.^22^

### Mechanistic interpretation

Visible light irradiation of **RuP3** generates RuP3*, which can in principle be quenched by two different mechanisms in the NiO|**RuP3**–Zr^4+^–**NiP** assembly: (a) oxidative quenching of RuP3* (*E*(**RuP3^+^**/RuP3*) ∼ –0.78 V *vs.* the normal hydrogen electrode (NHE), HOMO–LUMO gap *E*_H–L_(**RuP3**) = 2.19 eV, *λ*_em_ = 567 nm, Fig. S15†)^37^ by **NiP**, followed by hole injection from the oxidised **RuP3^+^** (*E*(**RuP3^+^**/**RuP3**) ∼ 1.41 V *vs.* NHE, Fig. S16†) into NiO (*E*_VB_(NiO) = 0.57 V *vs.* NHE at pH 3);^38^ or (b) reductive quenching of RuP3* by fast electron transfer from NiO into the HOMO of the dye to form the reduced species **RuP3^–^** (*E*(RuP3*/**RuP3^–^**) ∼ 0.80 V *vs.* NHE, Fig. 2b).^39^**RuP3^–^** has a sufficiently negative ground state reduction potential (*E*(**RuP3**/**RuP3^–^**) ∼ –1.29 V *vs.* NHE)^39^ to provide driving force for reduction of **NiP** (*E*(Ni^II/I^) = –0.35 V *vs.* NHE at pH 4.5).17*a* The reductive quenching mechanism for the NiO|**RuP3**–Zr^4+^–**NiP** assembly is much more likely since hole injection from a dye into NiO happens on very fast timescales of hundreds of femto- to picoseconds.^13^,15a Additionally, spectroscopic studies of a homogenous **RuP**–**NiP** system for photocatalytic H_2_ generation have shown previously that RuP* does not directly inject electrons into **NiP** in solution or when both molecules are anchored on ZrO_2_ as a non-injecting matrix. Reductive quenching by a SED or p-NiO yields **RuP^–^**, which was shown to efficiently reduce **NiP**.17*a* Reductive quenching of the NiO|**RuP3**–Zr^4+^–**NiP** system would provide the catalyst with an overpotential of ∼0.9 V (Fig. 2b).

Our results also suggest an important contribution of the Zr^4+^ interlayer to the efficiency of electron transfer away from the electrode surface to the associated catalyst. As Zr^4+^ is inert against reduction, it is unlikely that the Zr^4+^ coordinated to **RuP3** and **NiP** is acting as an electron relay similar to TiO_2_ in H_2_ evolving dye–TiO_2_–catalyst suspension systems.^18^ It is more likely that the Zr^4+^ layer prevents **NiP** from binding directly on the NiO surface in close proximity to NiO|**RuP3**, and that spatial separation of the reduced catalyst from the generated holes in NiO is beneficial to avoid charge recombination which is fast when dye and catalyst are in close proximity.^13^ Reports on the catalytic activity of photoanodes for water oxidation showed that stability was enhanced when the catalyst was spatially separated from the dye-sensitised electrode.25d,36a,^40^ Spectroscopic studies of a TiO_2_|**RuP3**–Zr^4+^–catalyst photoanode demonstrated that electron back transfer was slowed down significantly compared to a TiO_2_|catalyst electrode,25*b* which was assigned to increased spatial separation. Similar effects were also found for electron transfer between photoexcited TiO_2_ and cobaloxime catalysts with different linker lengths.^41^ These experiments show the high potential of using rationally designed dye–catalyst assemblies on photocathodes.

## Conclusions

A dye-sensitised photocathode with a co-immobilised nickel catalyst for light driven H_2_ generation in water has been reported. Co-deposition of the Ru dye **RuP3** and Ni catalyst **NiP** on the p-type semiconductor NiO in a supramolecular assembly of Zr^4+^-phosphonates provided us with a tool to control spatial arrangement of individual species without the need for elaborate chemical synthesis of dye–linker–catalyst dyads. This layer-by-layer approach keeps the catalyst in close proximity to the dye, but increases the distance to the semiconductor electrode. Our PEC experiments confirm that directed forward electron transfer from the excited dye to the catalyst is efficient and recombination kinetics are slowed down. The catalyst was therefore able to turn over and the hybrid electrode able to photo-generate H_2_ at an electrochemical underpotential, thereby demonstrating the potential of this system to store light in the chemical bonds of H_2_. Layer-by-layer assembly of dye and catalyst is therefore established as a novel strategy to produce molecule-based photocathodes for H_2_ evolution.

The efficiency of the presented system can be improved in the future by optimising the NiO|dye interface such as developing phosphonated push–pull dyes optimised for p-NiO,^12^ and fine-tuning of the H_2_ evolution catalyst. Further work will also include the synthesis and investigation of dyes, which are better suited to inject holes into the NiO valence band. Optimised molecule-containing photocathodes might ultimately become an attractive component for use in PEC cells for full water splitting.11d,^22^

## Experimental section

### Materials and methods

**NiP**,17*a***RuP**,^35^ and **RuP3** 24 (Fig. 1) were synthesised and characterised as previously reported. Chemicals for analytical measurements were purchased in the highest available purity and used without further purification. Ni(NO_3_)_2_·6H_2_O (Fisher Scientific, extra pure, 250 g) and hexamethylenetetramine (Sigma Aldrich, ReagentPlus, 99%) were used for the preparation of NiO electrodes. ITO nanopowder (Sigma Aldrich, particle size below 50 nm) was used for the preparation of mesostructured ITO electrodes. All electrochemical and analytical measurements were performed using Milli-Q^®^ H_2_O (*R* > 18 MΩ cm). ITO-covered glass sheets (Vision Tek Systems Ltd., *R* = 12 Ω cm^–2^, thickness 1.1 mm) were cut into 3 × 1 cm^2^ slides for preparation of working electrodes.

### Physical characterisation

An FEI Phillips XL30 SFEG SEM was used in the Electron Microscopy Suite in the Cavendish Laboratory, University of Cambridge. Powder X-ray diffraction (XRD) analysis was performed using a PANalytical BV X'Pert Pro X-ray diffractometer in the Department of Chemistry, University of Cambridge. XPS analysis was performed at the NEXUS XPS facility at Newcastle University. XPS spectra were calibrated to the C 1s signal at 284.8 eV. Quantification of Ni and Ru on the electrodes *via* ICP-OES (PerkinElmer Optima 2100 DV spectrometer) was carried out at the Department of Geography, University of Cambridge. High-resolution ATR-IR spectra were recorded on a Thermo Scientific Nicolet iS 50 FT-IR spectrometer with an ATR unit. UV-vis absorption spectra of electrodes were recorded in transmission mode in air, and UV-vis solution spectra were recorded in a quartz glass cuvette (1 cm path length) with a Varian Cary 50 UV-vis spectrophotometer. Fluorescence emission spectra of **RuP3** in Na_2_SO_4_ (0.1 M, pH 3) were measured with an Edinburgh Instruments FS5 spectrofluorometer.

### Electrochemical impedance spectroscopy

Electrochemical impedance spectroscopy (EIS) was performed on an IviumStat potentiostat in the frequency range of 100 kHz to 0.1 Hz in aqueous Na_2_SO_4_ solution (0.1 M, pH 3, 5 and 7). An equivalent circuit (inset Fig. S4†) consisting of electrolyte solution resistance (*R*_s_), constant phase element (CPE), and interfacial charge transfer resistance (*R*_CT_) was fitted to Nyquist plots obtained at different potentials in ZView® (Scribner Associates Inc.) to retrieve *C*_SC_ values required to form a Mott–Schottky plot. The Mott–Schottky equation, 
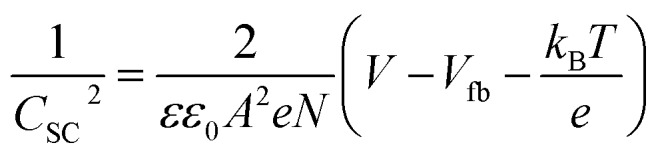
, where *C*_SC_ is the interfacial capacitance, *A* is the interfacial area, *N* the density of acceptors, *V* the applied potential, *V*_fb_ the flatband potential, *k*_B_ the Boltzmann constant, *T* the absolute temperature, and *e* the electronic charge, was used to calculate the flatband potential of NiO. 1/*C*_SC_^2^ plotted against applied potential results in a straight line with the intercept being equal to *V*_fb_ + *k*_B_*T*/*e*. The negative slope is indicative of the p-type character of the NiO electrodes.

### Preparation of nanostructured NiO electrodes26*b*

ITO covered glass slides (3 × 1 cm^2^) were cleaned by successive sonication in ethanol and acetone for 10 min, respectively. After drying in air, the conductive surface was confined to *ca.* 1 × 1.5 cm^2^ with electrical tape. The ITO glass slides were placed at approximately 45° with the conductive side facing down into separate vials and a solution of Ni(NiO_3_)_2_·6H_2_O and hexamethylenetetramine (250 mM each, 6 mL) was added to each vial. For hydrothermal growth of the films, the vials were placed into an oven (Thermo Scientific Heratherm) for 15, 30, 45 or 60 min at 90 °C and the reaction was stopped by the immediate addition of distilled water. The slides were rinsed with water, dried in air and the tape removed before the slides were annealed in a furnace (Carbolite) at 450 °C in air for 30 min (heating rate 20 °C min^–1^), and left to cool to room temperature in the furnace chamber.

### Preparation of mesostructured ITO electrodes^32^

ITO covered glass slides (3 × 1 cm^2^) were cleaned by heating them in a mixture of NH_3(conc.)_/H_2_O/H_2_O_2(30%)_ (1 : 5 : 1) at 70 °C for 30 min. The slides were rinsed with distilled water and dried in an oven at 180 °C for 1 h. The surface of the conductive side was confined with Scotch tape to circular 0.28 cm^2^. ITO nanopowder (20% w/w) was sonicated in EtOH (5 M acetic acid) for 1 hour and then doctor bladed on the ITO support. After drying in air, the tape was removed and the electrodes were annealed in a furnace (Carbolite) at 400 °C in air for 1 h (4 °C min^–1^ heating rate) and left to cool to room temperature in the oven chamber.

### Assembly of molecular photocathodes

The layer-by-layer deposition of dye (**RuP3** or **RuP**) and catalyst (**NiP**) was achieved by sequential immersion of the electrodes in solutions containing the molecular compounds at room temperature. NiO electrodes were submerged in solutions of **RuP3** or **RuP** (1 mM in H_2_O, overnight, washed with H_2_O and EtOH), ZrOCl_2_ (5 mM in EtOH (60% v/v in H_2_O), 2 h, washed with H_2_O and EtOH) and **NiP** (0.5 mM in MeOH, 2–3 h, washed with MeOH) to build up the supramolecular layered assemblies. The electrodes were dried in a stream of N_2_ after each immobilisation step. Multiple (*n*) catalyst and dye layers were assembled by repeating the immobilisation cycles *n* times. **RuP3** sensitised mesostructured ITO electrodes (ITO|**RuP3**) for controls were prepared according to the same procedure. The electrodes were stored in the dark prior use.

### Quantification of immobilised compounds on NiO

#### (A) Quantification of **RuP3** by UV-vis spectrophotometry

A NiO|**RuP3** electrode was immersed in aqueous NaOH (0.1 M, 1.0 mL) for 5 min to desorb the phosphonated species. The absorbance of the resulting solution at *λ* = 467 and 297 nm was used to determine the amount of **RuP3** detached from the surface (assuming quantitative detachment of phosphonic acids in pH 13 solution).

#### (B) Quantification of Ru, and Ni *via* ICP-OES

Surface-bound species were detached by immersing the electrodes in aqueous NaOH (0.1 M, 1.0 mL) for 5 min. The electrodes were rinsed with 10 mL H_2_O and then HNO_3_ (2% v/v) was added to a final volume of 25 mL. Samples were prepared and analysed from at least two individual electrodes for each condition and the averages are reported. Errors are given as maximum deviation from the average.

### Determination of **RuP3** oxidation potential

*E*(**RuP3^+^**/**RuP3**) was determined by recording a CV of **RuP3** in Na_2_SO_4_ (0.1 M, pH 3) with a boron doped diamond working, Ag/AgCl/KCl_(sat.)_ reference and Pt mesh counter electrode. The HOMO–LUMO gap *E*_H–L_(**RuP3**) was determined as the intersection of the normalised absorption and emission spectra of **RuP3** in Na_2_SO_4_ (0.1 M, pH 3).^24^*E*(**RuP3^+^**/RuP3*) was estimated by *E*(**RuP3^+^**/RuP3*) = *E*(**RuP3^+^**/**RuP3**) – *E*_H–L_(**RuP3**).

### Photoelectrochemistry

Electrochemical measurements were performed on an Ivium CompactStat potentiostat using a three-electrode configuration in a custom-made cell with a flat borosilicate window for PEC experiments. The prepared dye-sensitised NiO slides (1 cm^2^ geometrical surface area confined with electrical tape) were studied as working electrodes. A Pt mesh counter electrode, and a Ag/AgCl/KCl_(sat.)_ reference electrode were used in aqueous electrolyte solution (0.1 M Na_2_SO_4_, pH 3). Prior to any measurements, all solutions were purged for at least 10 min with N_2_ to remove O_2_. PEC experiments were performed under irradiation from the back-side of the electrodes with visible light (*λ* > 400 nm, UQG Optics filter) using a solar light simulator (Newport Oriel, 150 W, 100 mW cm^–2^) equipped with an AM1.5G and IR water filter.

CPPE was carried out in a custom-made 2-compartment PEC cell separated by a Nafion membrane (quartz glass window, 1.5 cm diameter). In the working compartment, 14.5 mL of electrolyte solution were used, leaving a gas headspace of 4.9 mL. In the counter compartment, 4.5 mL of electrolyte solution were used, leaving a gas headspace of 3.1 mL. Both compartments were purged prior to electrolysis with N_2_ containing CH_4_ (2%) as internal standard for gas chromatography measurements. The electrolyte solution (0.5 M Na_2_SO_4_, pH 3) in both compartments was stirred during the experiments. The amount of H_2_ in the headspace of the electrolysis cell was quantified by an Agilent 7890A Series gas chromatograph equipped with a 5 Å molecular sieve column. The GC oven temperature was kept constant at 45 °C, N_2_ was used as a carrier gas at an approximate flow rate of 3 mL min^–1^ and a thermal conductivity detector was used. In a standard experiment, the working electrode was held at the respective potential (*E*_appl_) in the dark for the first h, and after confirming the absence of headspace H_2_, it was exposed to visible light (*λ* > 400 nm, 100 or 200 mW cm^–2^) for 2 h at *E*_appl_. All CPPE experiments were carried out at least three times, unless noted otherwise. The mean values of the measurements are reported along with their standard errors.17*a*

## Supplementary Material

Supplementary information
