# Supplementary material for: Photoelectrochemical hydrogen production in water using a layer-by-layer assembly of a Ru dye and Ni catalyst on NiO
Source: Chem Sci. 2016 May 9;7(8):5537–46. doi: 10.1039/c6sc00715e (PMC6021778; doi:10.1039/c6sc00715e)
Supplement: Supplementary file 1 [file SC-007-C6SC00715E-s001.pdf]

Electronic Supplementary Material (ESI) for Chemical Science.
This journal is © The Royal Society of Chemistry 2016

Electronic Supporting Information

Photoelectrochemical hydrogen production in water using a layer-by-layer assembly of a Ru dye and Ni catalyst on NiO

Manuela A. Gross,[a] Charles E. Creissen,[a] Katherine L. Orchard,[a] Erwin Reisner[a*]

[a]Christian Doppler Laboratory for Sustainable SynGas Chemistry, Lensfield Road, CB2 1EW Cambridge, UK.

*Correspondence to: reisner@ch.cam.ac.uk

Table S1. Average pore size (measured as distance between individual sheets), film thickness, dye loading capacity and cathodic photocurrent after 100 s at E_{appl} = 0.3 V vs RHE in Na_2SO_4 (0.1 M, pH 3) in the presence of 4,4'-dithiodipyridine (DTDP, 1 mM) for NiO thin films after different growth times (see Experimental Section).

Growth time / min	Av. pore size / nm	Film thickness / µm	RuP3 loading / nmol cm^{-2}	$\|j\|$ / µA cm^{-2}
15	138 ± 33	1.15	5.3 ± 0.5	4.15 ± 0.17
30	290 ± 81	1.95	10.8 ± 0.8	8.79 ± 0.41
45	511 ± 107	3.01	13.0 ± 1.0	7.72 ± 0.14
60	605 ± 119	4.02	15.2 ± 1.0	6.47 ± 0.49

Table S2. Amounts of headspace H_2 gas evolved and Faradaic efficiency for all CPPE experiments (λ > 400 nm, 100 mW cm^{-2}, AM 1.5G). Unless otherwise noted, CPPE was carried out for 2 h and the background current was corrected for in the data analysis.

Composition	E_{appl} / V vs RHE	I / mW cm^{-2}	$n(H_2)$ / nmol [a]	FE / %
NiO|**RuP3**-Zr^{4+}-**NiP** [b]	0.5	100	6.78 ± 1.02	9.97 ± 2.48
NiO|**RuP3**-Zr^{4+}-**NiP** [b]	0.3	100	6.11 ± 0.68	8.63 ± 2.28
NiO|**RuP3**-Zr^{4+}-**NiP** [b]	0.3	200	6.99 ± 2.01	8.56 ± 3.19
NiO|**RuP3-NiP** [b]	0.3	100	H_2 traces [d]	–
NiO|**RuP3**-(Zr^{4+}-**NiP**)$_2$	0.5	100	5.11 ± 0.60	10.26 ± 2.18
NiO|**RuP3**-(Zr^{4+}-**NiP**)$_2$	0.3	100	6.78 ± 0.86	10.13 ± 1.82
NiO| (**RuP3**-Zr^{4+})$_2$-**NiP**	0.3	100	H_2 traces [d]	–
NiO|**RuP3**-Zr^{4+} [c]	0.0	100	no H_2	–
NiO	0.3	100	no H_2	–

[a] H_2 dissolved in the aqueous electrolyte was accounted for by using the Henry's law. [b] Values for $n(H_2)$ and FE determined after 1 h of irradiation. [c] Material degradation was observed during the course of the experiments (see Figure S14). [d] Amount of H_2 below limit of quantification.

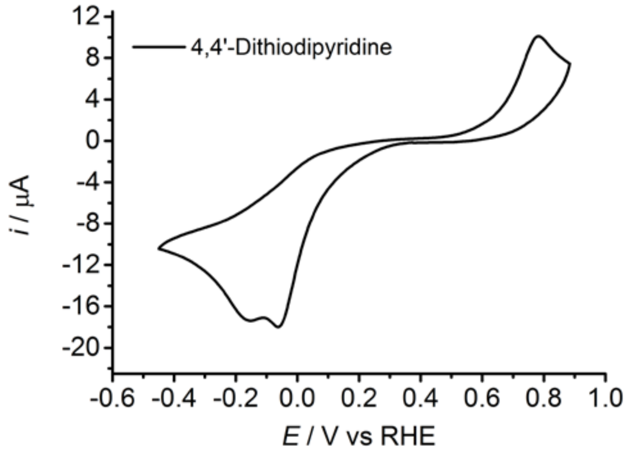

Figure S1. Cyclic voltamogramm of 4,4’-dithiodipyridine with a glassy carbon working electrode in an aqueous Na_2SO_4 solution (0.1 M, pH 3) at a scan rate 50 mV s^{-1} at room temperature.

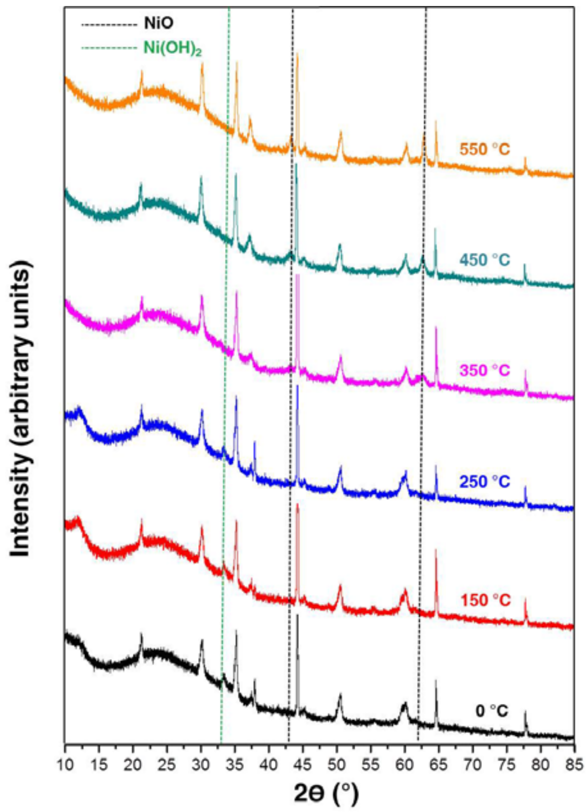

Figure S2. XRD pattern of hydrothermally grown $Ni(OH)_2$ thin films on ITO glass (ICSD collection code for ITO: 50847) after annealing for 30 min at the stated temperature. $Ni(OH)_2$ (green dotted line, ICSD collection code: 169978) converts to NiO (black dotted line, ICSD collection code: 9866) at temperatures from 350 °C. Reflections other than for $Ni(OH)_2$ or NiO can be attributed to the ITO support.

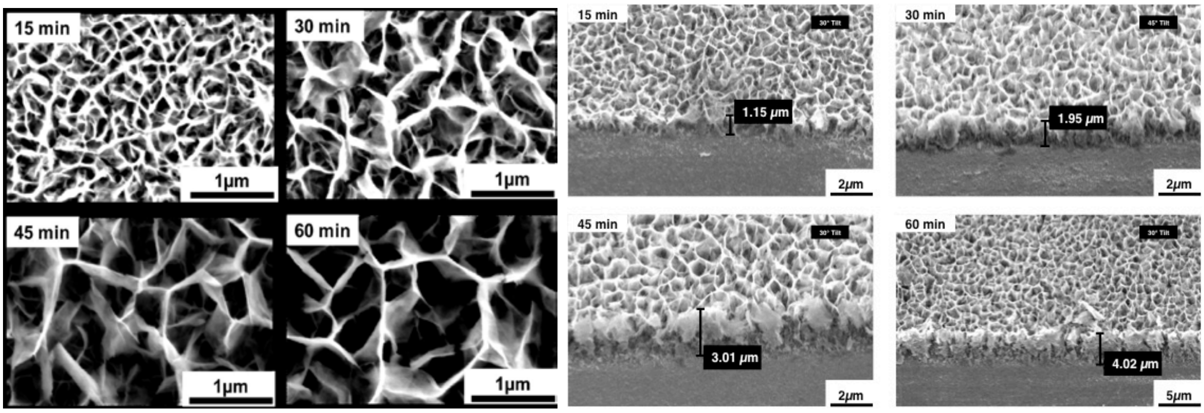

Figure S3. Top down (left panel) and tilted (right panel) SEM images showing morphology and thickness of NiO films after 15, 30, 45 and 60 min of hydrothermal growth at 90 °C and annealing in air at 450 °C.

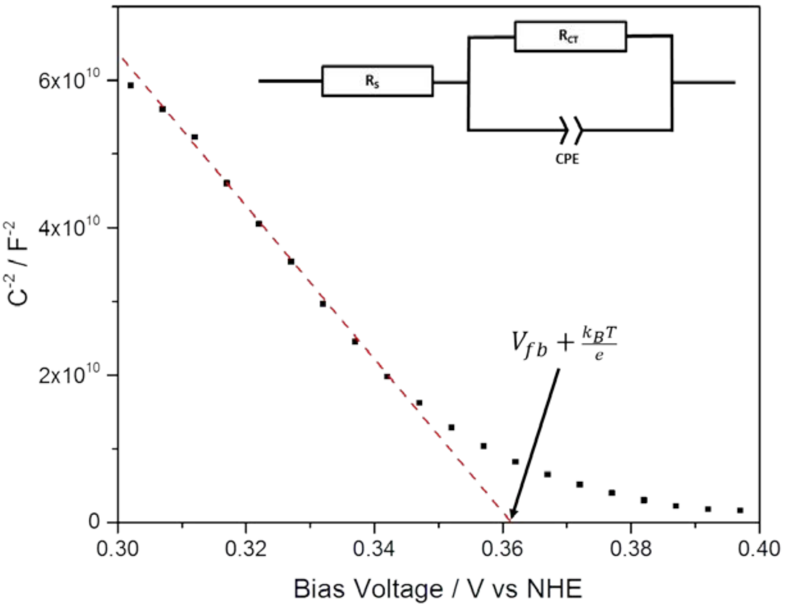

Figure S4. Mott-Schottky plot for a NiO electrode on ITO glass in aqueous Na_2SO_4 solution (0.1 M, pH 7) recorded under dark conditions at room temperature. Frequency ranged from 100 kHz to 0.1 Hz with an excitation voltage of 10 mV. Inset shows the equivalent circuit used to model the interface between semiconductor and electrolyte using ZView® (Scribner Associates Inc.).

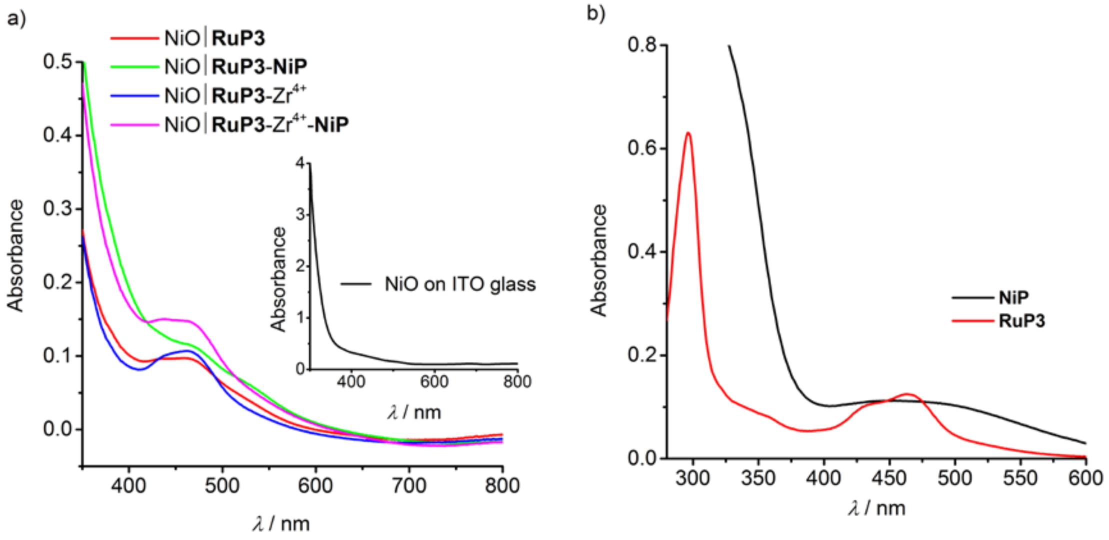

Figure S5. (a) UV-vis absorption spectra of NiO|**RuP3** electrodes (background absorption of NiO was subtracted from all spectra). Inset: UV-vis absorption spectrum of a NiO film (30 min hydrothermal growth) on ITO-coated glass. (b) Solution UV-vis spectra of **RuP3** ($1.33 \cdot 10^{-2}$ mM) in H_2O and **NiP** ($0.63 \cdot 10^{-2}$ mM) in Britton-Robinson buffer at pH 4 (low solubility of **NiP** prevented accurate measurements at pH 3). The spectra were recorded at room temperature.

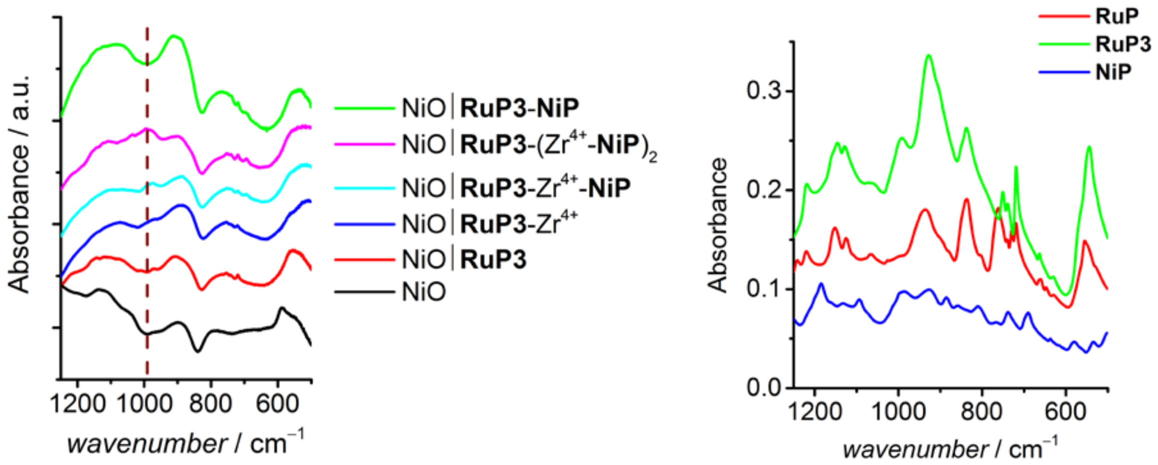

Figure S6. ATR-IR spectra of NiO electrodes (left) and of powdered samples of the compounds (right), showing the region of P=O and P–O stretching vibrations. The changes in the spectra are due to coordination of Zr^{4+} to the phosphonic acids (dashed line; see Hanson, K.; Torelli, D. A.; Vannucci, A. K.; Brennaman, M. K.; Luo, H.; Alibabaei, L.; Song, W.; Ashford, D. L.; Norris, M. R.; Glasson, C. R. K.; Concepcion, J. J.; Meyer, T. J. *Angew. Chem. Int. Ed.* 2012, ***51***, 12782-12785 for comparison).

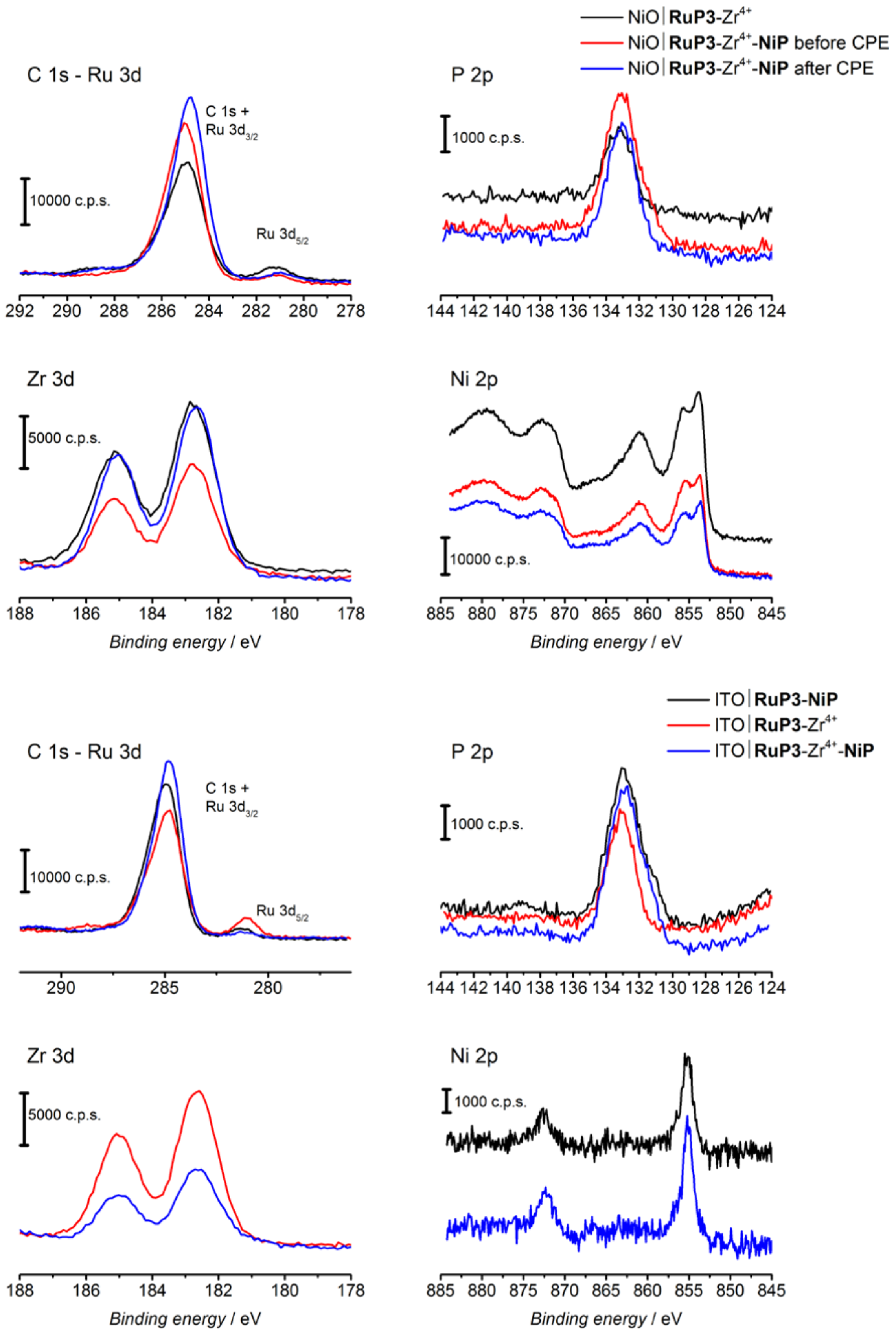

Figure S7. XPS analysis of different NiO (top) and ITO (bottom) hybrid electrodes.

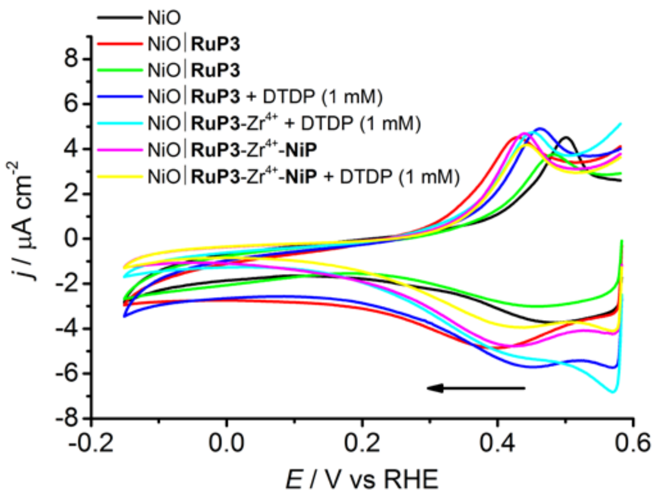

Figure S8. Cyclic voltammograms of NiO|**RuP3** electrodes in an aqueous Na_2SO_4 solution (0.1 M, pH 3) in the dark and in the presence or absence of soluble electron acceptor DTDP (1 mM) as a scan rate of 5 mV s^{-1} (the black arrow indicates the start of scan and scanning direction). No electrochemical response apart from the Ni^{2+}/Ni^{3+} surface oxidation was observed in the dark. The experiments were performed in a 3-electrode setup with Ag/AgCl/KCl$_{(sat.)}$ reference and Pt mesh counter electrodes under N_2 in a custom-made PEC cell at room temperature

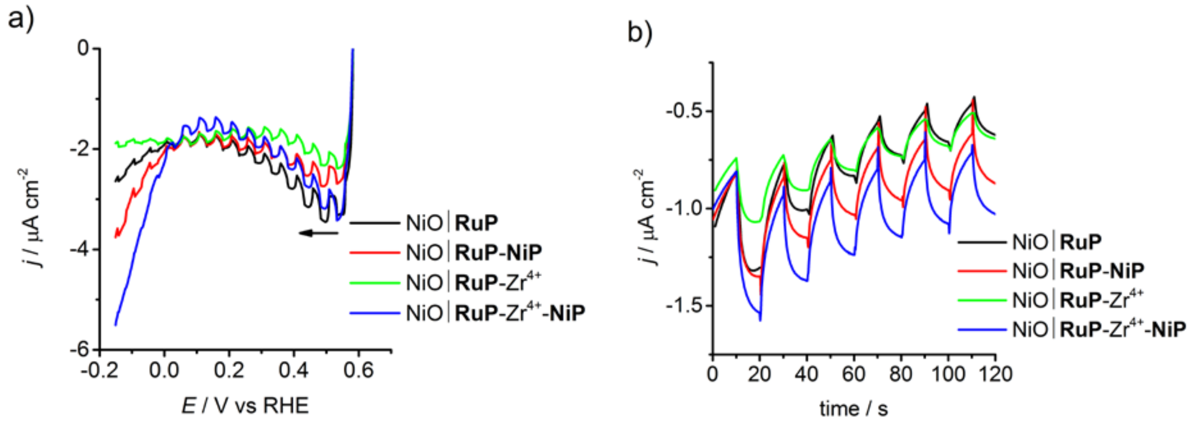

Figure S9. a) LSV (black arrow indicates scanning direction) and b) Chronoamperometry of NiO|**RuP** based electrodes at E_{appl} = 0.3 V vs RHE in an aqueous Na_2SO_4 solution (pH 3, 0.1 M) at room temperature. All experiments were performed in a 3-electrode setup with Ag/AgCl/KCl$_{(sat.)}$ reference and Pt mesh counter electrodes under N_2 in a custom-made photoelectrochemical cell at room temperature. UV-filtered light irradiation was standardised to 1 Sun (AM 1.5G filter, 100 mW cm^{-2} and $\lambda > 400$ nm).

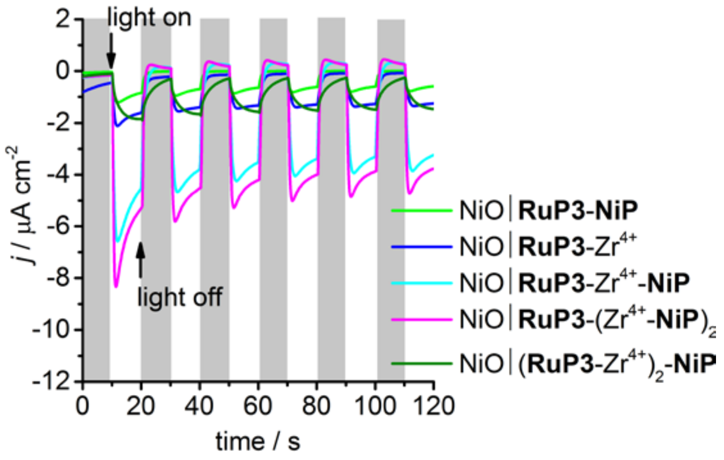

Figure S10. Chronoamperometry of NiO|**RuP3** based electrodes at E_{appl} = 0.5 V vs RHE under chopped light irradiation (λ > 400 nm, AM 1.5G filter, 100 mW cm^{-2}) in an aqueous Na_2SO_4 solution (pH 3, 0.1 M) at room temperature. All experiments were performed in a 3-electrode setup with Ag/AgCl/KCl$_{(sat.)}$ reference and Pt mesh counter electrodes under N_2 in a custom-made photoelectrochemical cell. These experiments were conducted at the end of a series of experiments at E_{appl} = 0.3 V and lower currents than with freshly prepared electrodes were observed.

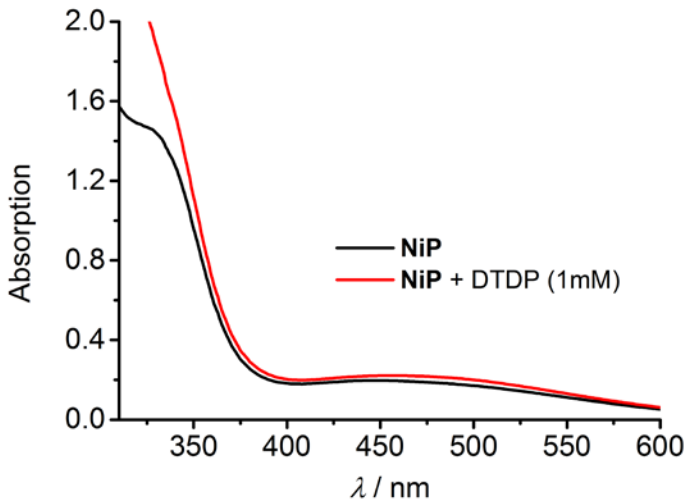

Figure S11. NiP (0.63·10^{-2} mM) in the absence and presence of DTDP in Britton-Robinson buffer (Britton, H. T. S.; Robinson, R. A. *J. Chem. Soc.* 1931, 1456-1462) at pH 4 and room temperature.

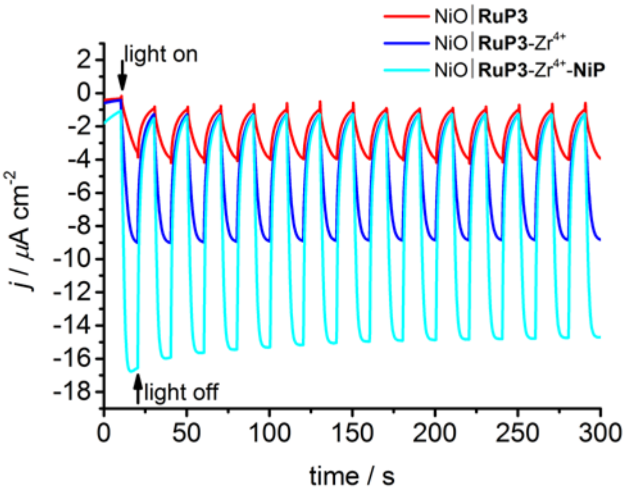

Figure S12. Chronoamperometry of NiO|**RuP3** based electrodes in the presence of DTDP at E_{appl} = 0.3 V vs RHE under chopped light irradiation (λ > 400 nm, AM 1.5G filter, 100 mW cm^{-2}) in an aqueous Na_2SO_4 solution (pH 3, 0.1 M) at room temperature. All experiments were performed in a 3-electrode setup with Ag/AgCl/$KCl_{(sat.)}$ reference and Pt mesh counter electrodes under N_2 in a custom-made photoelectrochemical cell. Light irradiation was standardised to 1 sun equivalent with AM 1.5G filter, 100 mW cm^{-2} and λ > 400 nm.

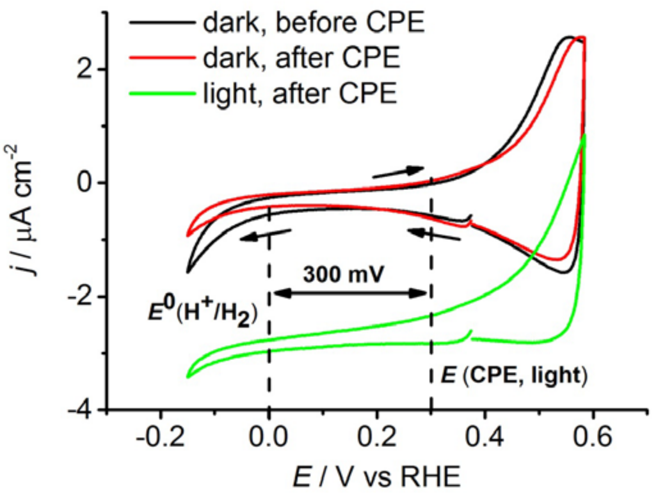

Figure S13. Cyclic voltamogramm in the dark and under visible light irradiation (λ > 400 nm, AM 1.5G filter, 100 mW cm^{-2}) of a NiO|**RuP3**-Zr^{4+}-**NiP** electrode before and after CPPE at E_{appl} = 0.3V vs RHE and room temperature. Black arrows indicate scanning direction, starting in reductive direction. Scan rate 5 mV s^{-1}. No material degradation was observed.

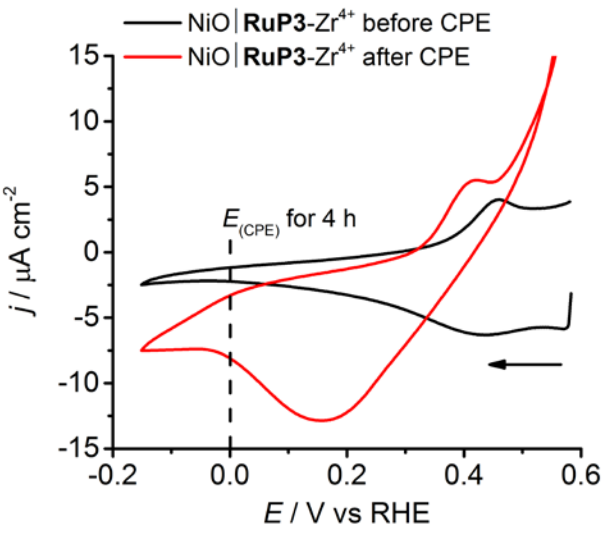

Figure S14. Cyclic voltamogramm in the dark of a NiO|**RuP3**-Zr^{4+} working electrode before and after CPPE under irradiation (λ > 400 nm, AM 1.5G filter, 100 mW cm^{-2}) at E_{appl} = 0.0 V vs RHE for 4 h at room temperature. Black arrows indicate the scanning direction, starting in reductive direction. Scan rate 5 mV s^{-1}. Material degradation in the absence of catalyst and soluble electron acceptor is observed.

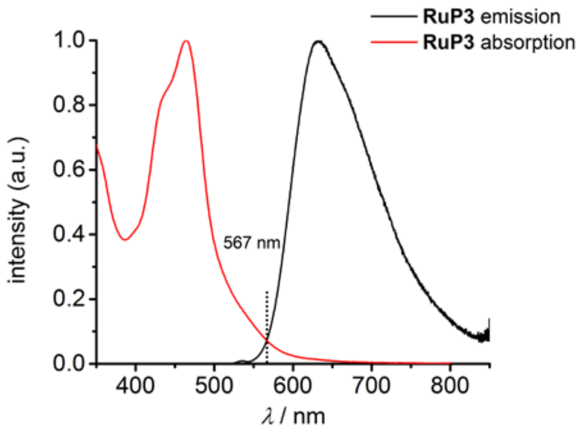

Figure S15. Normalised absorption and emission spectra of **RuP3** in Na_2SO_4 (0.1 M, pH 3).

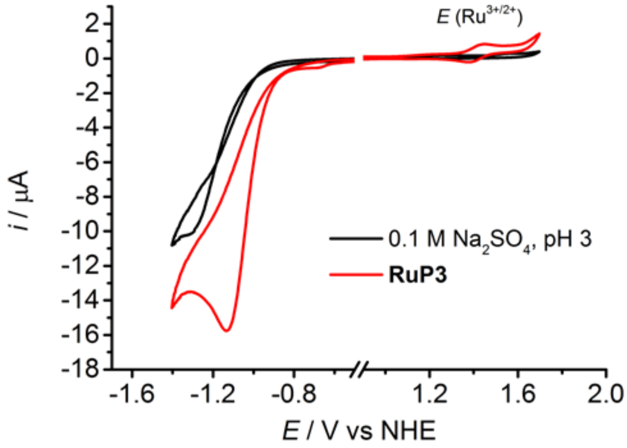

Figure S16. Cyclic voltamogramm of **RuP3** in Na_2SO_4 (0.1 M, pH 3) with a boron doped diamond working electrode, Ag/AgCl/$KCl_{(sat.)}$ reference and Pt mesh counter electrodes under N_2 at 50 mV s^{-1}.

End of Electronic Supporting Information
